# Changes in serious infection and mortality among patients with ANCA-associated vasculitis during the COVID-19 pandemic: an interrupted time-series analysis of J-CANVAS

**DOI:** 10.1016/j.ero.2026.03.013

**Published:** 2026-04-06

**Authors:** Satoshi Omura, Takashi Kida, Daiki Nakagomi, Yoshiyuki Abe, Makoto Wada, Naoho Takizawa, Atsushi Nomura, Yuji Kukida, Naoya Kondo, Hirosuke Takagi, Koji Endo, Shintaro Hirata, Naoto Azuma, Tohru Takeuchi, Shoichi Fukui, Kazuro Kamada, Ryo Yanai, Yusuke Matsuo, Yasuhiro Shimojima, Ryo Nishioka, Ryota Okazaki, Tomoaki Takata, Mayuko Moriyama, Ayuko Takatani, Yoshia Miyawaki, Tsuyoshi Shirai, Hiroaki Dobashi, Takafumi Ito, Isao Matsumoto, Toshihiko Takada, Toshiko Ito-Ihara, Nobuyuki Yajima, Takashi Kawaguchi, Kazuki Fujioka, Takahiro Seno, Masataka Kohno, Yutaka Kawahito

**Affiliations:** 1 Inflammation and Immunology, Graduate School of Medical Science, Kyoto Prefectural University of Medicine, Kyoto, Japan; 2 Department of Rheumatology, University of Yamanashi Hospital, Yamanashi, Japan; 3 Department of Internal Medicine and Rheumatology, Juntendo University, Tokyo, Japan; 4 Center for Rheumatic Disease, Japanese Red Cross Society Kyoto Daiichi Hospital, Kyoto, Japan; 5 Department of Rheumatology, Chubu Rosai Hospital, Aichi, Japan; 6 Immuno-Rheumatology Center, St. Luke’s International Hospital, Tokyo, Japan; 7 Department of Rheumatology, Japanese Red Cross Society, Kyoto Daini Hospital, Kyoto, Japan; 8 Department of Nephrology, Kyoto Katsura Hospital, Kyoto, Japan; 9 Department of Hematology and Rheumatology, Kagoshima University Hospital, Kagoshima, Japan; 10 Department of General Internal Medicine, Tottori Red Cross Hospital, Tottori, Japan; 11 Department of Clinical Immunology and Rheumatology, Hiroshima University Hospital, Hiroshima, Japan; 12 Department of Diabetes, Endocrinology and Clinical Immunology, Hyogo Medical University School of Medicine, Hyogo, Japan; 13 Department of Internal Medicine (IV), Osaka Medical and Pharmaceutical University, Osaka, Japan; 14 Department of Immunology and Rheumatology, Division of Advanced Preventive Medical Sciences, Nagasaki University Graduate School of Biomedical Sciences, Nagasaki, Japan; 15 Department of Rheumatology, Endocrinology and Nephrology, Faculty of Medicine and Graduate School of Medicine, Hokkaido University, Hokkaido, Japan; 16 Division of Rheumatology, Department of Medicine, Showa Medical University School of Medicine, Tokyo, Japan; 17 Department of Rheumatology, Tokyo Kyosai Hospital, Tokyo, Japan; 18 Department of Rheumatology, Graduate School of Medical and Dental Sciences, Institute of Science Tokyo (formerly Tokyo Medical and Dental University), Tokyo, Japan; 19 Department of Medicine (Neurology and Rheumatology), Shinshu University School of Medicine, Nagano, Japan; 20 Department of Nephrology and Rheumatology, Graduate School of Medical Science, Kanazawa University, Ishikawa, Japan; 21 Division of Respiratory Medicine and Rheumatology, Department of Multidisciplinary Internal Medicine, Faculty of Medicine, Tottori University, Tottori, Japan; 22 Division of Gastroenterology and Nephrology, Tottori University, Tottori, Japan; 23 Department of Rheumatology, Shimane University Faculty of Medicine, Shimane, Japan; 24 Rheumatic Disease Center, Sasebo Chuo Hospital, Nagasaki, Japan; 25 Department of Nephrology, Rheumatology, Endocrinology and Metabolism, Okayama University Graduate School of Medicine, Dentistry and Pharmaceutical Sciences, Okayama, Japan; 26 Department of Rheumatology, Tohoku University Hospital, Miyagi, Japan; 27 Division of Hematology, Rheumatology and Respiratory Medicine, Department of Internal Medicine, Faculty of Medicine, Kagawa University, Kagawa, Japan; 28 Division of Nephrology, Department of Internal Medicine, Teikyo University Chiba Medical Center, Chiba, Japan; 29 Department of Rheumatology, Institute of Medicine, University of Tsukuba, Ibaragi, Japan; 30 Department of General Medicine, Shirakawa Satellite for Teaching And Research (STAR) Fukushima Medical University, Fukushima, Japan; 31 The Clinical and Translational Research Center, University Hospital, Kyoto Prefectural University of Medicine, Kyoto, Japan; 32 Department of Healthcare Epidemiology, Kyoto University Graduate School of Medicine and Public Health, Kyoto, Japan; 33 Center for Innovative Research for Communities and Clinical Excellence, Fukushima Medical University, Fukushima, Japan; 34 Department of Clinical Assessment, Tokyo University of Pharmacy and Life Sciences, Tokyo, Japan

## Abstract

**Objectives:**

In antineutrophil cytoplasmic antibody (ANCA)-associated vasculitis, infection risk exceeds that of the general population, and infection is a leading cause of death. However, the impact of the COVID-19 pandemic on serious infections and mortality in this population remains uncertain.

**Methods:**

This observational study used data from the nationwide registry in Japan (Japan Collaborative Registry of ANCA-Associated Vasculitis), which includes patients with newly diagnosed and relapsed ANCA-associated vasculitis from 29 sites between January 2017 and March 2023. The primary outcome was serious infection requiring hospitalisation; the secondary outcome was all-cause mortality. The impact of COVID-19 was estimated using an interrupted time-series analysis, with the boundary between April and May 2020 as the change point, following the first declaration of a state of emergency in Japan. Monthly incidence rates were modelled using segmented Poisson regression. Rate ratios (RRs) for level and slope changes with 95% CI were reported. Several sensitivity analyses were performed.

**Results:**

Among 1064 patients, total follow-up was 37,535 person-months; 205 serious infections and 96 deaths occurred. For serious infection, the level-change RR was 0.54 (95% CI: 0.31-0.94) and the slope-change RR was 1.01 (0.98-1.04). For all-cause mortality, the level-change RR was 0.35 (0.12-0.98) and the slope-change RR was 1.001 (0.94-1.06). Findings were robust across sensitivity analyses.

**Conclusions:**

Immediately after the onset of the COVID-19 pandemic, a clear reduction in serious infections and mortality was observed without major changes in patient characteristics. These findings may reflect the impact of social and personal infection control measures.

**WHAT IS ALREADY KNOWN ON THIS TOPIC**

• Seasonal infectious diseases declined in the general population during the COVID-19 pandemic, but whether similar changes occurred in serious infections and mortality among patients with antineutrophil cytoplasmic antibody (ANCA)-associated vasculitis was unknown.

**WHAT THIS STUDY ADDS**

• In a nationwide Japanese registry (Japan Collaborative Registry of ANCA-Associated Vasculitis), serious infections and all-cause mortality in ANCA-associated vasculitis declined immediately after the onset of the COVID-19 pandemic.

**HOW THIS STUDY MIGHT AFFECT RESEARCH, PRACTICE OR POLICY**

• Optimising the timing and intensity of infection-prevention behaviours may help improve prognosis in ANCA-associated vasculitis, and this should be tested in future studies.

Alt-text: Unlabelled box dummy alt text

## INTRODUCTION

In 2019, COVID-19, caused by SARS-CoV-2 [1], spread worldwide, leading to major social and healthcare disruptions. For patients with autoimmune diseases receiving immunosuppressive therapy, the pandemic posed particular challenges; in UK real-world database studies, patients with autoimmune rheumatic diseases had higher risks than the general population of COVID-19-related hospitalisation and death and, early in the pandemic, of all-cause mortality [2,3].

By contrast, the general population in multiple countries experienced declines in seasonal infectious diseases, including influenza, during the pandemic [[4], [5], [6]]. Hospitalisations for non–COVID-19 pneumonia and other respiratory conditions, such as asthma and chronic obstructive pulmonary disease, also decreased [7,8]. These contrasting patterns—heightened susceptibility to COVID-19 in immunosuppressed patients vs reduced incidence of other respiratory infections in the general population—prompt questions about how overall infection and mortality among patients with autoimmune diseases have changed relative to the prepandemic period. This question is particularly pertinent to antineutrophil cytoplasmic antibody (ANCA)-associated vasculitis, in which infection risk exceeds that of the general population [9], and infection is a leading cause of death [10]. In this study, we hypothesised that the declines in community-acquired infections observed in the general population—driven by both individual- and societal-level infection control measures—may have similarly occurred among patients with ANCA-associated vasculitis.

We therefore used a nationwide, multicentre registry to assess changes in serious infections and all-cause mortality among patients with ANCA-associated vasculitis before and during the COVID-19 pandemic, applying an interrupted time series (ITS) design.

## METHODS

### Study design and patients

We conducted a quasiexperimental ITS study [11] using the Japan Collaborative Registry of ANCA-Associated Vasculitis (J-CANVAS). The registry enrols adults (≥20 years) with newly diagnosed or relapsing ANCA-associated vasculitis from 29 participating sites in Japan. Patients diagnosed between January 2017 and March 2023 were retrospectively identified and followed up through March 2024. Disease subtype was classified as microscopic polyangiitis (MPA), granulomatosis with polyangiitis (GPA), or eosinophilic GPA (EGPA) according to the 2012 Chapel Hill Consensus Conference definitions [12] and the European Medicines Agency algorithm [13].

Because enrolment in 2017 was sparse, the analytic time series comprised monthly observations from January 2018 through March 2024. All patients with MPA, GPA, or EGPA registered during this period were included. To minimise bias from early hospitalisation-related infections, we excluded the first month of person-time after treatment initiation, when most patients were hospitalised. Community-acquired infections are uncommon; endogenous infections, particularly cytomegalovirus activations, are common [14].

### Data collection

All clinical data were retrospectively abstracted at each participating site from medical records. Baseline characteristics before treatment initiation included demographics (age, sex, height, and weight) and laboratory measures (serum creatinine and ANCA serotype: myeloperoxidase-ANCA, proteinase 3-ANCA, or seronegative). Sex was recorded from medical records as documented at each participating site; gender identity was not collected. Treatment information included glucocorticoid dose (expressed as prednisolone equivalents), rituximab, cyclophosphamide, avacopan, mepolizumab, and trimethoprim–sulfamethoxazole prophylaxis. During follow-up, outcomes captured were serious infections requiring hospitalisation or prolongation of hospitalisation, and all-cause mortality. All data were entered into a centralised electronic data capture system (Viedoc; PCG Solutions).

### Intervention

We defined the intervention point at the boundary between April and May 2020, and considered May 2020 onwards as the COVID-19 pandemic period, based on Japan’s first declaration of a state of emergency in April 2020 [15]. These noncoercive soft-lockdown measures were accompanied by border controls, active case finding and contact tracing through public health centres, progressive digital reporting, and coordinated public health messaging, collectively leading to substantial reductions in population mobility and mixing.

### Outcomes

The primary outcome was serious infection, defined as infection requiring hospitalisation or prolonging hospitalisation. The secondary outcome was all-cause mortality.

Accordingly, the study period was divided into January 2018 to April 2020 (prepandemic) and May 2020 to March 2024 (during pandemic). For mortality analyses, June 2020 was used as the intervention point, based on the prespecified assumption that pandemic-related changes in individual- and societal-level infection control measures would begin to affect the incidence of serious infection after approximately 1 month, with mortality outcomes expected to follow after an additional 1-month lag.

### Statistical analysis

**Figure 1:**
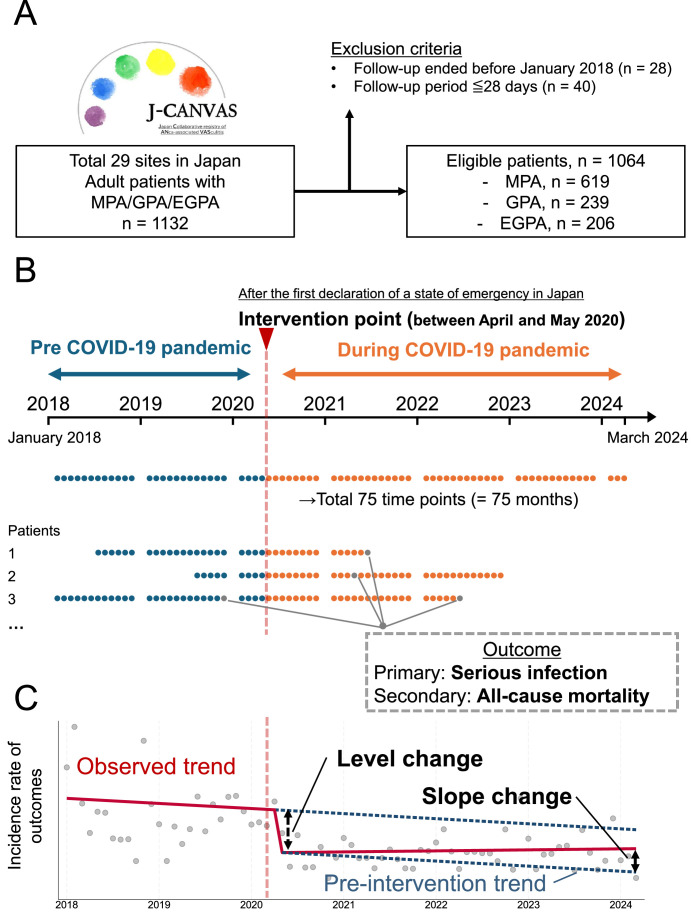
Cohort assembly and study timeline, with intervention point and impact model. (A) Flow of patients in the J-CANVAS registry. Adults with MPA, GPA, or EGPA were enrolled from 29 sites in Japan. At-risk person-time began on day 29 after treatment initiation; patients with total follow-up ≤28 days contributed no at-risk time and were excluded. (B) Schematic illustration of the study design. Each patient’s follow-up was divided into consecutive months, and monthly incidence rates (events per person-month) were calculated for serious infection (primary outcome) and all-cause mortality (secondary outcome) across 75 monthly time points. The intervention point was set at the boundary between April and May 2020, corresponding to Japan’s first declaration of a state of emergency; months from May 2020 onwards were treated as the COVID-19 pandemic period. (C) Conceptual impact model for the interrupted time-series analysis. Monthly incidence rates were modelled using segmented Poisson regression with a log (person-months) offset, estimating the preintervention trend, the immediate level change at the intervention point, and the change in slope during the COVID-19 pandemic period. EGPA, eosinophilic granulomatosis with polyangiitis; GPA, granulomatosis with polyangiitis; J-CANVAS, Japan Collaborative Registry of ANCA-Associated Vasculitis; MPA, microscopic polyangiitis.

We employed a quasiexperimental ITS framework with monthly time steps to estimate the association between the COVID-19 pandemic and incidence rates of outcomes. An overview of the study design is shown in Figure 1B. We prespecified an impact model comprising an immediate step (level) change at the intervention point and a during-pandemic slope (Fig 1C) [16,17]. Monthly incidence rates (per person-month) were modelled using segmented Poisson regression with a log (person-months) offset. To address overdispersion and residual autocorrelation, we scaled the Pearson *χ*^²^ by the residual degrees of freedom. Seasonality was accounted for using Fourier sine/cosine pairs, which provide a flexible and parsimonious representation of recurring seasonal patterns (order selected based on residual plots and autocorrelation function/partial autocorrelation function diagnostics) [18]. We reported rate ratios (RRs) for level and slope changes with 95% CI and displayed observed, fitted, and counterfactual (projected prepandemic trend) series. Several sensitivity analyses were conducted. First, we analysed a negative-control outcome, loss to follow-up from the registry, using the same ITS specification [19]. Second, we shifted the intervention point by ±2 months and estimated the corresponding level and slope changes using the same ITS specification as in the primary analysis. Third, we fit a model with 2 intervention points, adding the boundary between May and June 2023 (the end of universal COVID-19 case reporting in Japan) as a second intervention point. Fourth, we estimated multivariable segmented Poisson models, additionally adjusting for monthly mean glucocorticoid dose and renal function (estimated glomerular filtration rate [eGFR]). Finally, we report results from all model specifications, including those with scale adjustment for overdispersion, seasonal adjustment using Fourier terms. Data were summarised as mean (SD) or absolute numbers (percentages). All analyses were performed using Stata version 19 (StataCorp LLC).

### Patient and public involvement

No patients and/or public were involved in the planning or launch of this registry (J-CANVAS), nor in the design, analysis, or manuscript of this study.

## RESULTS

### Epidemic context in Japan during the COVID-19 pandemic

Japan confirmed its first COVID-19 case on January 15, 2020. In April 2020, the government issued the first declaration of a state of emergency, followed by 2 additional declarations in January and April 2021. Over the subsequent 3 years, Japan experienced 8 epidemic waves, with the seventh and eighth waves during the Omicron period being the largest. By the time the government downgraded the legal status of COVID-19 and ended universal all-case surveillance on May 7, 2023, approximately 34 million confirmed cases and 74,688 deaths had been reported nationwide [20].

### Patients and outcomes

Of 1132 patients enrolled in J-CANVAS, 1064 (MPA, n = 619; GPA, n = 239; EGPA, n = 206) were included in the analysis (Fig 1A). Across January 2018–March 2024, the monthly cumulative registry counts summed to 38,419, and the total follow-up time was 37,535.0 person-months. Patient characteristics by calendar period (with 2020 split into prepandemic and during pandemic segments) are summarised in Table 1. A detailed month-by-month count of registered patients is presented in Supplementary Table S1. Around the intervention point (the boundary between April and May 2020), there were 28 prepandemic and 47 during-pandemic monthly time points. During follow-up, 205 serious infections, 96 deaths, and 236 losses to follow-up were observed. Outcome counts by period are shown in Table 2.

**Table 1:** Characteristics and treatment in each year before and during the COVID-19 pandemic

	Year							Total
	Prepandemic			During pandemic				
	2018/1–2018/12	2019/1–2019/12	2020/1–2020/4	2020/5–2020/12	2021/1–2021/12	2022/1–2022/12	2023/1–2024/3	
No. of patients	342	501	510	604	678	759	737	1064
Cumulative number of patients	2903	4530	1886	4241	6990	7903	9966	38,419
Age, y	69.2 (14.1)	69.8 (14.0)	70.3 (13.7)	70.5 (13.6)	70.7 (13.6)	71.3 (13.3)	71.7 (13.2)	70.8 (13.5)
Female	1672 (57.6)	2649 (58.5)	1080 (57.3)	2462 (58.1)	4101 (58.7)	4661 (59.0)	5896 (59.2)	22521 (58.6)
MPA	1630 (56.1)	2414 (53.3)	1026 (54.4)	2267 (53.5)	3645 (52.1)	4105 (51.9)	5111 (51.3)	20,198 (52.6)
GPA	720 (24.8)	1199 (26.5)	475 (25.2)	1071 (25.3)	1854 (26.5)	2052 (26.0)	2543 (25.5)	9914 (25.8)
EGPA	553 (19.0)	917 (20.2)	385 (20.4)	903 (21.3)	1491 (21.3)	1746 (22.1)	2312 (23.2)	8307 (21.6)
MPO-ANCA	2106 (72.5)	3239 (71.5)	1376 (73.0)	3084 (72.7)	5063 (72.4)	5718 (72.4)	7171 (72.0)	27,757 (72.2)
PR3-ANCA	415 (14.3)	708 (15.6)	285 (15.1)	646 (15.2)	1057 (15.1)	1175 (14.9)	1449 (14.5)	5735 (14.9)
eGFR, mL/min/1.73m^2^	57.1 (24.8)	57.6 (23.5)	56.6 (22.5)	55.5 (22.8)	56.3 (21.7)	58.7 (23.8)	61.6 (24.3)	58.3 (23.5)
PSL dose, mg	11.7 (8.4)	9.6 (7.7)	8.6 (6.9)	8.1 (6.3)	7.0 (5.6)	6.1 (5.4)	4.9 (4.1)	7.2 (6.3)
RTX^a^	688 (23.7)	1204 (26.6)	515 (27.3)	1208 (28.5)	2066 (29.6)	2531 (32.0)	3533 (35.5)	11,745 (30.6)
IVCY^b^	838 (28.9)	925 (20.4)	354 (18.8)	696 (16.4)	1024 (14.6)	1046 (13.2)	1142 (11.5)	6025 (15.7)
Avacopan	8 (0.3)	7 (0.2)	0	0	4 (0.1)	79 (1.0)	630 (6.3)	728 (1.9)
Mepolizumab	23 (0.8)	112 (2.5)	72 (3.8)	210 (5.0)	467 (6.7)	728 (9.2)	1302 (13.1)	2914 (7.6)
AZA	785 (27.0)	1287 (28.4)	559 (29.6)	1259 (29.7)	2067 (29.6)	2263 (28.6)	2952 (29.6)	11172 (29.1)
MTX	122 (4.2)	276 (6.1)	122 (6.5)	262 (6.2)	479 (6.9)	579 (7.3)	653 (6.6)	2493 (6.5)
MMF	47 (1.6)	144 (3.1)	65 (3.4)	174 (4.1)	364 (5.2)	520 (6.6)	700 (7.0)	2011 (5.2)

ANCA, antineutrophil cytoplasmic antibody; AZA, azathioprine; eGFR, estimated glomerular filtration rate; EGPA, eosinophilic granulomatosis with polyangiitis; GPA, granulomatosis with polyangiitis; IVCY, intravenous cyclophosphamide; MMF, mycophenolate mofetil; MPA, microscopic polyangiitis; MPO, myeloperoxidase; MTX, methotrexate; PR3, proteinase 3; PSL, prednisolone; RTX, rituximab.

Continuous variables are shown as mean (SD); categorical variables are shown as number (%). Patient characteristics and treatments were presented as cumulative monthly case counts within each period.

a Within 24 weeks after the last RTX use.

b Within 12 weeks after the last IVCY use.

**Table 2:** The details of outcomes in each year before and during the COVID-19 pandemic

	Year							Total
	Prepandemic			During pandemic				
	2018/1-2018/12	2019/1-2019/12	2020/1-2020/4	2020/5-2020/12	2021/1-2021/12	2022/1-2022/12	2023/1-2024/3	
Cumulative follow-up, mo	2798.6	4406.4	1829.3	4172.3	6891.6	7798.5	9638.3	37,535.0
Serious infection, n (IR)	26 (11.1)	34 (9.3)	14 (9.2)	16 (4.6)	27 (4.7)	44 (6.8)	44 (5.5)	205 (6.6)
Bacterial (respiratory)	11 (4.7)	16 (4.4)	4 (2.6)	5 (1.4)	9 (1.6)	8 (1.2)	16 (2.0)	69 (2.2)
Bacterial (nonrespiratory)	5 (2.1)	8 (2.2)	6 (3.9)	3 (0.9)	8 (1.4)	13 (2.0)	14 (1.8)	57 (1.8)
COVID-19	0	0	0	0	2 (0.3)	15 (2.3)	10 (1.3)	27 (0.9)
PCP	2 (0.9)	4 (1.1)	2 (1.3)	2 (0.6)	0	2 (0.3)	0	12 (0.4)
CMV	2 (0.9)	1 (0.3)	1 (0.7)	2 (0.6)	2 (0.3)	1 (0.2)	1 (0.1)	10 (0.3)
Others	6 (2.6)	5 (1.4)	1 (0.7)	4 (1.2)	6 (1.0)	5 (0.8)	3 (0.4)	30 (1.0)
Mortality, n (IR)	8 (3.4)	13 (3.5)	8 (5.2)	9 (2.6)	13 (2.3)	17 (2.6)	28 (3.5)	96 (3.1)
Loss-to-follow-up, n (IR)	33 (14.1)	36 (9.8)	17 (11.1)	38 (10.9)	34 (5.9)	32 (4.9)	46 (5.7)	236 (7.5)

CMV, cytomegalovirus; IR, incidence rate; ITS, interrupted time series; PCP, pneumocystis pneumonia.

Counts and incidence rates are presented for each outcome. Incidence rates are expressed per 100 person-years; monthly counts were used for ITS analysis. For serious infections, event counts are additionally shown by infection type.

### Main results—changes in serious infections and all-cause mortality during the COVID-19 pandemic from the ITS models

**Figure 2:**
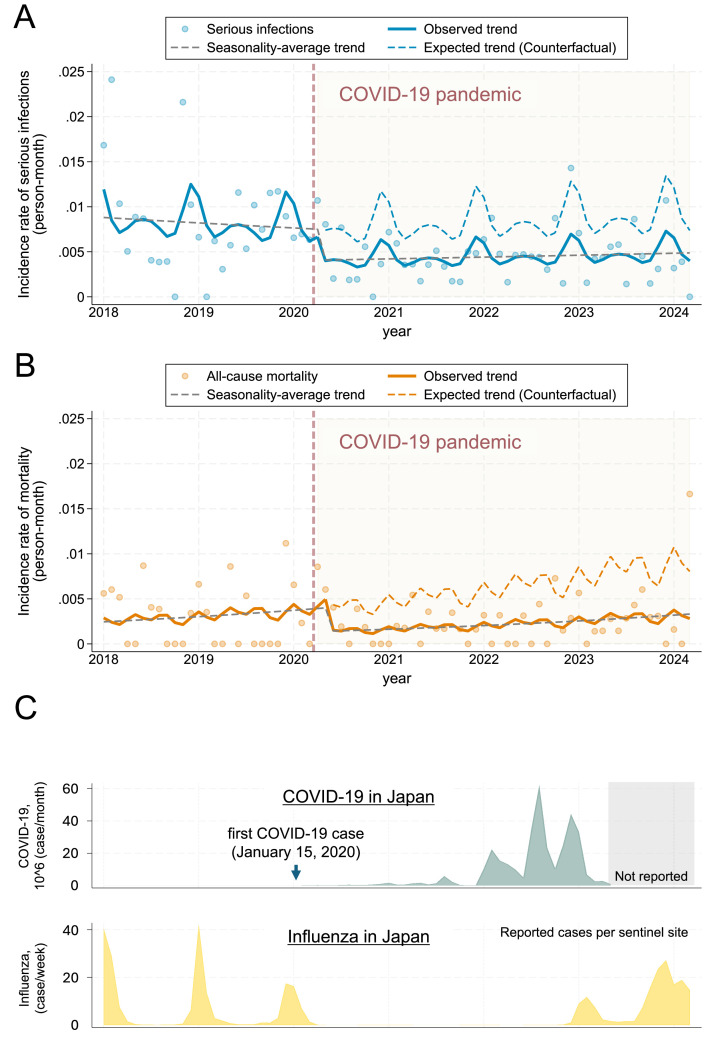
Trends in incidence rates of serious infection and mortality from interrupted time-series analysis. Incidence‐rate trends for (A) serious infections and (B) all-cause mortality were estimated using an interrupted time-series design. Each panel shows the modelled counterfactual trend extrapolated from the prepandemic period (navy dashed curve) alongside the observed trend (orange solid curve). Point estimates for the level and slope changes are summarised in Table 3. Panel C displays the national incidence of COVID-19 and seasonal influenza in Japan; COVID-19 figures are from all-case reporting [21], whereas influenza reflects the national average from sentinel (physician-based) surveillance [22].

**Figure 3:**
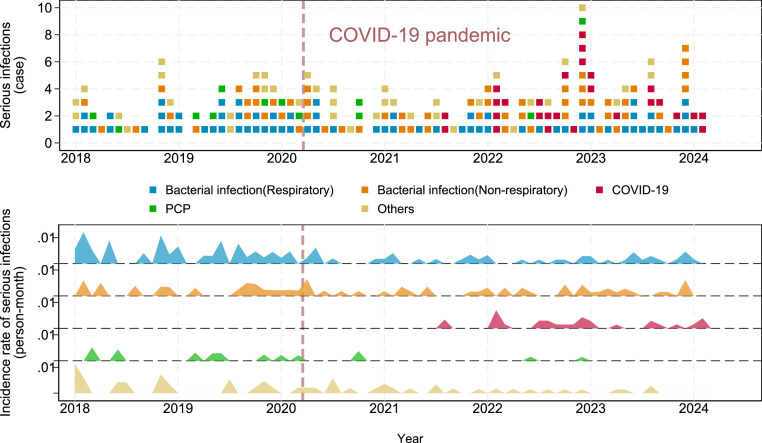
The counts and incidence rates in each infection type. Upper panel: monthly counts of serious infections by infection type; each box denotes 1 event. Lower panel: monthly incidence rates by infection type, expressed per person-month. Event types are shown as bacterial respiratory, bacterial nonrespiratory, PCP, COVID-19, and others. CMV, cytomegalovirus; PCP, pneumocystis pneumonia.

Observed incidence trends are presented alongside the model-based counterfactual from the prepandemic period; for context, national incidence of COVID-19 [21] and seasonal influenza [22] in Japan are also plotted (Fig 2) [21,22]. For the primary outcome (serious infection), the RR for the level change was 0.54 (95% CI: 0.31-0.94), and the RR for the slope change was 1.01 (95% CI: 0.98-1.04). For the secondary outcome (all-cause mortality), the level-change RR was 0.35 (95% CI: 0.12-0.98) and the slope-change RR was 1.001 (95% CI: 0.94-1.06). For both outcomes, incidence declined immediately and remained below prepandemic levels through March 2024. These estimates are summarised in Table 3. Infection-type–specific counts and changes in incidence are shown in Figure 3. In descriptive patterns, bacterial respiratory infections appeared to trend downwards, whereas nonrespiratory bacterial infections were generally stable over time.

**Table 3:** Level change and slope change of outcomes during the COVID-19 pandemic

	Level change		Slope change	
	RR	95% CI	RR	95% CI
Serious infection	0.54	0.31-0.94	1.01	0.98-1.04
Mortality	0.35	0.12-0.98	1.001	0.94-1.06
Loss-to-follow-up (Negative control)	0.98	0.54-1.78	0.998	0.96-1.03

RR, rate ratio.

The intervention point was set to the boundary between April and May 2020 for serious infections and loss to follow-up, and to the boundary between May and June 2020 (with a 1-mo lag) for mortality. Estimates were obtained from segmented Poisson regression models, including a scale parameter ×2 and adjustment for seasonality using Fourier terms. Results from all other model specifications are provided in Supplementary Table S5.

### Sensitivity analyses—change in negative-control outcome (loss to follow-up)

Applying the same ITS framework with loss to follow-up as a negative-control outcome yielded a level-change RR of 0.98 (95% CI: 0.54-1.78) and a slope-change RR of 0.998 (95% CI: 0.96-1.03) (Table 3), suggesting a low risk of spurious detection for observed trends in serious infections and mortality.

### Sensitivity analyses—alternative lags around the intervention point

Results from alternative intervention-point specifications (−2, −1, +1, +2 months around May 2020 for infections and June 2020 for mortality) are provided in Supplementary Table S2. The largest deviations from the counterfactual were observed at the main intervention point (or within ±1 month), with attenuated effects at ±2 months.

### Sensitivity analyses—defining a second intervention point

A second intervention point (June 2023) was specified, and level- and slope-change estimates at each point were obtained from the ITS models. For the primary outcome (serious infection), the level-change RR at the first intervention point was 0.49 (95% CI: 0.27-0.89) and the slope-change RR was 1.02 (95% CI: 0.98-1.05); at the second intervention point, the corresponding RRs were 1.14 (95% CI: 0.46-2.84) and 0.93 (95% CI: 0.80-1.07). For all-cause mortality, the level- and slope-change RRs at the first intervention point were 0.40 (95% CI: 0.13-1.21) and 0.99 (95% CI: 0.93-1.06), and at the second intervention point, the level- and slope-change RRs were 0.92 (95% CI: 0.20-4.21) and 1.07 (95% CI: 0.86-1.33) (Supplementary Table S3). For both outcomes, the first intervention point (May 2020) showed the same pattern as the primary analysis, with an immediate decline and a trajectory that remained stable during the pandemic, although a clear level change was not observed for all-cause mortality. At the second intervention point (June 2023), neither the level nor the slope showed a clear change.

### Sensitivity analyses—covariate adjustment within the ITS models

In the primary ITS models, monthly mean oral prednisolone dose and monthly mean eGFR were included as covariates, each separately and jointly, and the level and slope changes were re-estimated. The resulting estimates were qualitatively consistent with those from the primary analysis (Supplementary Table S4).

### Comparison across all model specifications

All models used during dispersion/seasonality tuning are compared in Supplementary Table S5, and no material deviations from the primary results were observed.

## DISCUSSION

In our ITS analysis, the onset of the COVID-19 pandemic was associated with an immediate downwards level change in the rates of serious infection and all-cause mortality among patients with ANCA-associated vasculitis, with no clear difference in the during-pandemic slope, suggesting that the reductions were sustained over time. These patterns were consistent across outcome model specifications, and no intervention-related shift was detected for the negative-control outcome (loss to follow-up), supporting the robustness of the primary findings.

Immediately after the onset of the COVID-19 pandemic, the level change in serious infections we observed paralleled the contemporaneous decline in seasonal influenza in Japan. As shown in Figure 2, the near disappearance of influenza activity in Japan after the onset of the COVID-19 pandemic has been previously reported [23], and similar reductions in seasonal infections, including influenza, have been documented in other regions [[4], [5], [6]]. It has been reported that both individual-level preventive behaviours such as masking and hand hygiene, as well as societal infection control measures, reduce non–COVID-19 pneumonia [7,24,25]. Moreover, patients with rheumatic diseases adopted stricter isolation measures than the general population early in the pandemic [26]. Taken together, stronger personal and social preventive practices may have contributed to the observed reduction in serious infections among patients with ANCA-associated vasculitis. Given that infection is a leading cause of death in ANCA-associated vasculitis [10], the decline in mortality may largely reflect the reduction in infections. Other explanations for the level change should also be considered. As shown in Table 1, the average prescribed dose of prednisolone declined over time. Because a reduced-dose glucocorticoid regimen was reported in 2020 (the PEXIVAS trial [Plasma Exchange and Glucocorticoids for Treatment of Anti-Neutrophil Cytoplasm Antibody–Associated Vasculitis]) [27], we evaluated potential confounding by glucocorticoid dose. In multivariable ITS models that adjusted for monthly mean prednisolone dose (and, separately, renal function), estimates were similar to the primary analysis (Supplementary Table S3), suggesting a limited influence of dosing changes on the observed level change. Consistent with this, our international comparison conducted in 2020 indicated that glucocorticoid tapering in Japan proceeded more slowly than the reduced-dose protocol, implying that widespread uptake of the regimen may have been limited at that time [28].

During the pandemic period, we observed no meaningful change in the during-pandemic slope of infection incidence. This pattern contrasts with trends in the general population; as shown in Figure 2, seasonal influenza activity re-emerged from late 2022, whereas infection incidence among patients with ANCA-associated vasculitis appeared to remain suppressed. The finding persisted when we specified June 2023 as a second intervention point, a period corresponding to the end of universal COVID-19 case reporting in May 2023 and a broader relaxation of population-level mitigation, with similar estimated effects (Supplementary Table S2). One plausible explanation is the continuation of infection-prevention behaviours. Prior work reported that patients with inflammatory arthritis maintained stricter isolation measures than the general population early in the pandemic [29]. If these individual-level precautions largely underpinned the initial level change, their continuation could likewise account for the sustained reduction in infections. Another potential contributor is increased uptake of non–COVID-19 vaccines. In Japan, an increase in the distribution volumes of pneumococcal vaccines has been reported following the COVID-19 pandemic, which may have also contributed to the observed decrease in infections [30]. At the same time, evolving treatment practice may also have contributed. For patients with MPA and GPA, the LoVAS trial (Low-dose Glucocorticoids Vasculitis Induction Study) provided further support for a lower-dose glucocorticoid regimen [31], and avacopan enabled more advanced glucocorticoid-tapering/-free approaches [32]. In patients with EGPA, mepolizumab facilitates glucocorticoid tapering [33]. In our cohort, the monthly prednisolone dose decreased over time, and the use of avacopan and mepolizumab increased (Table 1), suggesting that changes in therapy may also have contributed to the reduction in infections.

This study reinforces that mitigating infections is central to improving outcomes in patients with ANCA-associated vasculitis. However, social isolation measures during the pandemic were associated with worsening mental health among individuals with rheumatic diseases [34]. Therefore, more intensive infection-prevention interventions should be implemented in a targeted and time-limited manner, eg, strengthening precautions for selected patients during periods of heightened susceptibility. We previously reported that hypogammaglobulinemia was associated with subsequent serious infections in ANCA-associated vasculitis [35]; such markers could help identify at-risk states in which temporarily augmenting preventive measures may be warranted. The effectiveness and feasibility of these targeted strategies should be evaluated in prospective studies.

A key strength of this study is the use of a large-scale, nationwide, multicentre cohort for a rare disease and a narrowly defined patient population, combined with a quasiexperimental ITS approach that strengthens causal inference. Together, these features enabled a rigorous evaluation and demonstrated clear, sustained pandemic-associated changes in outcomes.

This study had several limitations. ITS analysis assumes no major concurrent changes in the underlying patient population other than the intervention. Because our registry accrues patients sequentially, we cannot fully exclude the possibility that shifts unrelated to the pandemic contributed to the observed changes in outcomes. For example, pandemic-era alterations in admission thresholds and bed shortages could have reduced hospital-treated serious infections; however, such shifts would not readily explain the concurrent decrease in mortality. Furthermore, the negative-control analysis using loss to follow-up with the same ITS specification showed no intervention-related level or slope change, arguing against increased during-pandemic attrition, particularly selective loss of more severe cases, as an explanation for the observed decline in serious infections and mortality. Taken together, these checks make it less likely that unanticipated changes in the registry population alone account for the findings. Transportability is another limitation. Our results reflect the situation in Japan, where the direct mortality burden of COVID-19 in the general population was comparatively lower than in many other settings [36]. Reports from Europe early in the pandemic (2020-2021) indicated that patients with systemic vasculitis, including ANCA-associated vasculitis, had a higher risk of severe COVID-19 outcomes [37]. In regions with a greater direct impact of COVID-19, a reduction in all-cause mortality similar to that observed here may not occur. Whether similar patterns are observed in other populations warrants evaluation in external cohorts.

## Conclusion

Using an ITS design, we observed an immediate level change and a sustained reduction in serious infections and all-cause mortality among patients with ANCA-associated vasculitis during the COVID-19 pandemic. The magnitude of these changes appears clinically meaningful. Future studies should assess whether similar reductions can be achieved through targeted infection-prevention behaviours and other feasible measures in routine care.

## Competing interests

SO has received speaker’s fees from Chugai, UCB, and Janssen, and support for attending meetings from AbbVie. TK has received speaker’s fees from Asahi Kasei, Eisai, and Mitsubishi Tanabe. DN has received a research grant from Asahi Kasei, and speaker’s fees from Asahi Kasei, Kissei, and Chugai. YK has received speaker’s fees from Chugai and GlaxoSmithKline. HT has received speaker’s fees from Asahi Kasei, Astellas, and Kissei, and supports for attending meetings from Asahi Kasei, Chugai, and Kissei. SH has received a speaker’s fee from Chugai, Kissei, Glaxo SmithKline, and has received a research grant from Chugai. NA has received a research grant from Asahi Kasei and Chugai, and speaker’s fees from Asahi Kasei, Bristol Myers Squibb, Mitsubishi Tanabe, and Pfizer. RN has received a speaker’s fee from Asahi Kasei. TT has received research grants from Kyowa Kirin and Mitsubishi Tanabe, and a speaker’s fee from Kyowa Kirin. MM has received speaker’s fees from Asahi Kasei and Mitsubishi Tanabe. TI has received speaker’s fees from Kyowa Kirin, serves as a board member of the Japanese Society for Therapeutics and Engineering, the Japanese Society for Psychonephrology, and the International Society for Apheresis, and serves as Vice-President of the Japanese Society for Apheresis. IM has received research grants from Asahi Kasei, Chugai, and Mitsubishi Tanabe, and speaker’s fees from Asahi Kasei, Astellas, Boehringer Ingelheim, Bristol Myers Squibb, Chugai, GlaxoSmithKline, Eli Lilly, Mitsubishi Tanabe, Pfizer, and UCB. HD has received speaker’s fees from Kissei, GlaxoSmithKline, Chugai, and research funding from Chugai. TI-I has received a speaker’s fee from Kissei. KF has received speaker’s fees from AbbVie, Asahi Kasei Pharma, AstraZeneca, Chugai, Eisai, Eli Lilly, Gilead Sciences, Janssen (Johnson & Johnson), Mitsubishi Tanabe Pharma, Pfizer, Sanofi, and UCB. TS has received speaker’s fees from Asahi Kasei, AstraZeneca, AbbVie, Boehringer Ingelheim, Bristol Myers Squibb, Chugai, Eisai, GlaxoSmithKline, Janssen, Kissei, Kyorin, Mitsubishi Tanabe, Taisho, Pfizer, and UCB, and a research grant from UCB. YK has received research grants from Asahi Kasei and Chugai, and speaker’s fees from Asahi Kasei, Chugai, GSK, Pfizer, Novartis, Kissei, and Viatris. All the other authors declare they have no competing interests.

## Appendix Supplementary materials

Image, application 1

## Footnotes

Supplementary material associated with this article can be found in the online version at doi:10.1016/j.ero.2026.03.013.
