# Supplementary material for: Changes in serious infection and mortality among patients with ANCA-associated vasculitis during the COVID-19 pandemic: an interrupted time-series analysis of J-CANVAS
Source: EULAR Rheumatol Open. 2026 Apr 6;2(2):100165. doi: 10.1016/j.ero.2026.03.013 (PMC13425167; doi:10.1016/j.ero.2026.03.013)
Supplement: Supplementary file 1 [file mmc1.docx]

**Title:** Changes in serious infection and mortality among patients with ANCA-associated vasculitis during the COVID-19 pandemic: an interrupted time-series analysis of J-CANVAS

**Supplementary Table S1. Monthly number of patients by disease type**

**Supplementary Table S2** **Alternative lags models around the intervention point**

**Supplementary Table S3. Two intervention-points models**

**Supplementary Table S4. Covariate adjustment models**

**Supplementary Table S5. Comparison across all model specifications**

**Supplementary Table S1. Monthly number of patients by disease type**

| Calendar year | Total | MPA | GPA | EGPA |
| --- | --- | --- | --- | --- |
| January 2018 | 179 | 101 | 46 | 32 |
| February 2018 | 188 | 107 | 48 | 33 |
| March 2018 | 201 | 113 | 50 | 38 |
| April 2018 | 214 | 121 | 51 | 42 |
| May 2018 | 232 | 133 | 54 | 45 |
| June 2018 | 241 | 135 | 59 | 47 |
| July 2018 | 251 | 144 | 58 | 49 |
| August 2018 | 262 | 148 | 65 | 49 |
| September 2018 | 268 | 151 | 66 | 51 |
| October 2018 | 279 | 155 | 71 | 53 |
| November 2018 | 292 | 161 | 75 | 56 |
| December 2018 | 296 | 161 | 77 | 58 |
| January 2019 | 308 | 164 | 83 | 61 |
| February 2019 | 315 | 166 | 86 | 63 |
| March 2019 | 327 | 173 | 88 | 66 |
| April 2019 | 338 | 179 | 92 | 67 |
| May 2019 | 352 | 187 | 95 | 70 |
| June 2019 | 358 | 187 | 97 | 74 |
| July 2019 | 380 | 200 | 101 | 79 |
| August 2019 | 402 | 211 | 108 | 83 |
| September 2019 | 420 | 227 | 109 | 84 |
| October 2019 | 436 | 235 | 113 | 88 |
| November 2019 | 443 | 239 | 113 | 91 |
| December 2019 | 451 | 246 | 114 | 91 |
| January 2020 | 457 | 251 | 116 | 90 |
| February 2020 | 466 | 252 | 119 | 95 |
| March 2020 | 475 | 257 | 119 | 99 |
| April 2020 | 488 | 266 | 121 | 101 |
| May 2020 | 502 | 276 | 124 | 102 |
| June 2020 | 510 | 276 | 127 | 107 |
| July 2020 | 528 | 283 | 130 | 115 |
| August 2020 | 533 | 285 | 133 | 115 |
| September 2020 | 532 | 284 | 135 | 113 |
| October 2020 | 538 | 287 | 136 | 115 |
| November 2020 | 547 | 288 | 141 | 118 |
| December 2020 | 551 | 288 | 145 | 118 |
| January 2021 | 552 | 289 | 146 | 117 |
| February 2021 | 555 | 291 | 146 | 118 |
| March 2021 | 563 | 297 | 148 | 118 |
| April 2021 | 570 | 299 | 151 | 120 |
| May 2021 | 577 | 303 | 151 | 123 |
| June 2021 | 575 | 299 | 152 | 124 |
| July 2021 | 585 | 304 | 156 | 125 |
| August 2021 | 591 | 308 | 159 | 124 |
| September 2021 | 592 | 309 | 159 | 124 |
| October 2021 | 601 | 312 | 160 | 129 |
| November 2021 | 610 | 315 | 162 | 133 |
| December 2021 | 619 | 319 | 164 | 136 |
| January 2022 | 623 | 320 | 165 | 138 |
| February 2022 | 624 | 323 | 164 | 137 |
| March 2022 | 631 | 329 | 164 | 138 |
| April 2022 | 631 | 328 | 164 | 139 |
| May 2022 | 643 | 333 | 168 | 142 |
| June 2022 | 659 | 339 | 174 | 146 |
| July 2022 | 669 | 347 | 174 | 148 |
| August 2022 | 678 | 356 | 174 | 148 |
| September 2022 | 679 | 354 | 175 | 150 |
| October 2022 | 682 | 357 | 174 | 151 |
| November 2022 | 688 | 357 | 178 | 153 |
| December 2022 | 696 | 362 | 178 | 156 |
| January 2023 | 702 | 361 | 179 | 162 |
| February 2023 | 702 | 362 | 179 | 161 |
| March 2023 | 711 | 364 | 182 | 165 |
| April 2023 | 711 | 364 | 183 | 164 |
| May 2023 | 709 | 363 | 182 | 164 |
| June 2023 | 702 | 361 | 181 | 160 |
| July 2023 | 695 | 358 | 179 | 158 |
| August 2023 | 687 | 354 | 176 | 157 |
| September 2023 | 678 | 348 | 173 | 157 |
| October 2023 | 667 | 342 | 169 | 156 |
| November 2023 | 660 | 338 | 168 | 154 |
| December 2023 | 651 | 333 | 166 | 152 |
| January 2024 | 635 | 326 | 162 | 147 |
| February 2024 | 590 | 304 | 147 | 139 |
| March 2024 | 466 | 233 | 117 | 116 |

EGPA: eosinophilic granulomatosis with polyangiitis, GPA: granulomatosis with polyangiitis, MPA: microscopic polyangiitis

**Supplementary Table S2** **Alternative lags models around the intervention point**

| Change point lag | change point | Level change | | Slope change | |
| --- | --- | --- | --- | --- | --- |
|  |  | RR | 95%CI | RR | 95%CI |
| Serious Infections |  |  |  |  |  |
| –2 months | March 2020 | 0.69 | 0.39, 1.21 | 1.01 | 0.98, 1.05 |
| –1month | April 2020 | 0.66 | 0.37, 1.17 | 1.01 | 0.98, 1.05 |
| Main analysis | May 2020 | 0.54 | 0.31, 0.94 | 1.01 | 0.98, 1.04 |
| +1 month | June 2020 | 0.50 | 0.29, 0.85 | 1.01 | 0.98, 1.04 |
| +2 months | July 2020 | 0.58 | 0.34, 0.99 | 1.02 | 0.99, 1.05 |
| All-cause mortality |  |  |  |  |  |
| –2 months | April 2020 | 0.73 | 0.23, 2.30 | 1.01 | 0.94, 1.08 |
| –1 month | May 2020 | 0.44 | 0.15, 1.27 | 0.9996 | 0.94, 1.07 |
| Main analysis | June 2020 | 0.35 | 0.12, 0.98 | 1.001 | 0.94, 1.06 |
| +1 month | July 2020 | 0.31 | 0.11, 0.86 | 1.004 | 0.95, 1.06 |
| +2 months | August 2020 | 0.33 | 0.12, 0.90 | 1.01 | 0.96, 1.07 |

RR: rate ratio; CI: confidence interval.

**Supplementary Table S3. Two intervention-points models**

|  | Level change | | Slope change | |
| --- | --- | --- | --- | --- |
|  | RR | 95%CI | RR | 95%CI |
| Serious Infections |  |  |  |  |
| point 1  (2020/5~) | 0.49 | 0.27, 0.89 | 1.02 | 0.98, 1.05 |
| point 2  (2023/6~) | 1.14 | 0.46, 2.84 | 0.93 | 0.80, 1.07 |
| Mortality |  |  |  |  |
| point 1  (2020/6~) | 0.40 | 0.13, 1.21 | 0.99 | 0.93, 1.06 |
| point 2  (2023/6~) | 0.92 | 0.20, 4.21 | 1.07 | 0.86, 1.33 |

RR: rate ratio; CI: confidence interval.

**Supplementary Table S4. Covariate adjustment models**

|  | Level change | | Slope change | |
| --- | --- | --- | --- | --- |
|  | RR | 95%CI | RR | 95%CI |
| Serious Infections |  |  |  |  |
| adjusted for prednisolone dosage | 0.50 | 0.29, 0.86 | 0.98 | 0.94, 1.02 |
| adjusted for eGFR | 0.26 | 0.10, 0.67 | 1.05 | 0.999, 1.11 |
| adjusted for prednisolone dosage and eGFR | 0.31 | 0.12, 0.78 | 1.01 | 0.94, 1.09 |
| All-cause mortality |  |  |  |  |
| Unadjusted Poisson | 0.35 | 0.15, 0.80 | 1.0001 | 0.95, 1.05 |
| adjusted for prednisolone dosage | 0.35 | 0.12, 0.98 | 0.997 | 0.92, 1.08 |
| adjusted for eGFR | 0.52 | 0.12, 2.20 | 0.97 | 0.87, 1.08 |
| adjusted for prednisolone dosage and eGFR | 0.56 | 0.13, 2.36 | 0.95 | 0.83, 1.09 |

RR: rate ratio; CI: confidence interval.

**Supplementary Table S5. Comparison across all model specifications**

|  | Level change | | Slope change | |
| --- | --- | --- | --- | --- |
|  | RR | 95%CI | RR | 95%CI |
| Serious Infections |  |  |  |  |
| Unadjusted Poisson | 0.52 | 0.30, 0.91 | 1.01 | 0.98, 1.04 |
| Poisson, with scale parameter set to ×2 to account for overdispersion | 0.52 | 0.30, 0.92 | 1.01 | 0.98, 1.04 |
| Poisson, adjusted for overdispersion, and seasonality (K=1) | 0.57 | 0.33, 0.98 | 1.01 | 0.98, 1.04 |
| Poisson, adjusted for overdispersion, and seasonality (K=2) | 0.54 | 0.31, 0.92 | 1.01 | 0.98, 1.04 |
| Poisson, adjusted for overdispersion, and s  seasonality (K=3) | 0.54 | 0.31, 0.94 | 1.01 | 0.98, 1.04 |
| All-cause mortality |  |  |  |  |
| Unadjusted Poisson | 0.35 | 0.15, 0.80 | 1.0001 | 0.95, 1.05 |
| Poisson, with scale parameter set to ×2 to account for overdispersion | 0.35 | 0.12, 0.98 | 1.001 | 0.94, 1.06 |
| Poisson, adjusted for overdispersion, and seasonality (K=1) | 0.35 | 0.13, 0.95 | 1.001 | 0.95, 1.06 |
| Poisson, adjusted for overdispersion, and seasonality (K=2) | 0.34 | 0.12, 0.93 | 1.0001 | 0.94, 1.06 |
| Poisson, adjusted for overdispersion, and seasonality (K=3) | 0.35 | 0.12, 0.98 | 1.001 | 0.94, 1.06 |

RR: rate ratio; CI: confidence interval.
